# Improved Risk Prediction of Acute Myocardial Infarction in Patients With Stable Coronary Artery Disease Using an Amino Acid-Assisted Model

**DOI:** 10.1155/2024/9935805

**Published:** 2024-08-30

**Authors:** Yi-Jing Zhao, Yong Li, Feng-Xiang Wang, Hao Lv, Yaoyao Qu, Lian-Wen Qi, Pingxi Xiao

**Affiliations:** ^1^ State Key Laboratory of Natural Medicines School of Traditional Chinese Pharmacy China Pharmaceutical University, Nanjing, China; ^2^ Department of Cardiology Pukou Hospital of Chinese Medicine Affiliated to China Pharmaceutical University, Nanjing, China; ^3^ Department of Cardiology The Affiliated Wujin Hospital of Jiangsu University, Changzhou, China; ^4^ The Clinical Metabolomics Center China Pharmaceutical University, Nanjing, China; ^5^ Department of Cardiology The Fourth Affiliated Hospital of Nanjing Medical University, Nanjing, China

**Keywords:** acute myocardial infarction, amino acid, coronary artery disease, methylated amino acid, risk prediction

## Abstract

Patients with stable coronary artery disease (CAD) are at an increased risk of acute myocardial infarction (AMI), particularly among older individuals. Developing a reliable model to predict AMI occurrence in these patients holds the potential to expedite early diagnosis and intervention. This study is aimed at establishing a circulating amino acid-assisted model, incorporating amino acid profiles alongside clinical variables, to predict AMI risk. A cohort of 874 CAD patients from two independent centers was analyzed. Plasma amino acid levels were quantified using liquid chromatography tandem mass spectrometry (LC-MS/MS) employing a targeted metabolomics approach. This methodology incorporated ^13^C isotope-labeled internal standards for precise quantification of 27 amino acids. Univariate logistic regression was applied to identify differentially expressed amino acids that distinguished between stable CAD and AMI patients. To assess prediction performance, receiver operating characteristic (ROC) curve and nomogram analyses were utilized. Five amino acids—lysine, methionine, tryptophan, tyrosine, and N6-trimethyllysine—emerged as potential biomarkers (*p* < 0.05), exhibiting significant differences in their expression levels across the two centers when comparing stable CAD with AMI patients. For AMI risk prediction, the base model, utilizing 12 clinical variables, achieved areas under the curve (AUC) of 0.7387 in the discovery phase (*n* = 623) and 0.8205 in the external validation set (*n* = 251). Notably, the integration of these five amino acids into the prediction model significantly enhanced its performance, increasing the AUC to 0.7651 in the discovery phase (Delong's test, *p* = 1.43e‐02) and to 0.8958 in the validation set (Delong's test, *p* = 8.91e‐03). In conclusion, the circulating amino acid-assisted model effectively enhances the prediction of AMI risk among CAD patients, indicating its potential clinical utility in facilitating early detection and intervention.

## 1. Introduction

Acute myocardial infarction (AMI) is a critical manifestation of coronary artery disease (CAD) in aging individuals, and it remains a leading cause of global morbidity and mortality [1–3]. Given its significant impact, early and accurate detection of AMI is crucial for initiating prompt medical intervention [4]. Clinically, AMI is typically categorized into ST-segment elevation myocardial infarction and non-ST-elevation myocardial infarction [5]. Patients with stable CAD are particularly vulnerable to the development of AMI. While cardiac troponin T serves as the gold standard biomarker for AMI, its increased levels can also be attributed to other conditions such as heart failure, myocarditis, chest trauma, and renal failure [6]. This lack of specificity in the early stages of AMI underscores the urgent need for novel biomarkers with higher sensitivity and specificity to enhance risk prediction and aid in earlier diagnosis.

Abnormal amino acid metabolism has been linked to atherosclerosis development. Branched-chain amino acids (valine, leucine, and isoleucine), homocysteine, tryptophan, and its major catabolite kynurenine have been closely associated with cardiac events [7–11]. N-Methylated amino acids, derivatives with one to three methyl substituents at the amine group, have emerged as novel metabolic biomarkers for various diseases. For instance, N-methylglycine (sarcosine) has been identified as a metabolic intermediary in cancer cell invasion, with elevated levels correlating with prostate cancer progression [12]. Similarly, trimethyllysine, a precursor of trimethylamine N-oxide (TMAO), has shown promising results in predicting short- and long-term outcomes in patients with acute coronary syndromes [13]. However, the specific roles of these amino acids, particularly their methylated analogs, in the risk prediction and pathogenesis of AMI remain poorly understood and require further investigation.

The ongoing discovery of biomarkers remains an invaluable complement to clinical disease risk assessment [14–16]. Clinical metabolomics, a rapidly evolving field within system biology, focuses on identifying biomarkers by analyzing alterations in metabolite levels associated with various diseases. In our previous research, we employed an untargeted metabolomics approach to uncover potential biomarkers for CAD in a substantial cohort of 2324 participants [17]. Among the perturbed metabolic pathways identified, disturbances in amino acid metabolism stood out as significant. However, untargeted metabolomics provides a broad overview of metabolite levels without precise quantification. For this reason, a targeted strategy for accurate amino acid quantification in CAD patients is imperative. In this work, we developed a targeted metabolomics approach and a 5-amino acid-assisted prediction model to improve AMI risk prediction. This approach holds the potential to significantly contribute to the improvement of risk assessment and disease management in CAD patients.

## 2. Materials and Methods

### 2.1. Study Population

The CAD patients recruited from Center 1 (the Affiliated Wujin Hospital of Jiangsu University) between August 2013 and December 2015 formed the discovery phase. Patients enrolled from Center 2 (the Sir Run Run Hospital of Nanjing Medical University) between April 2017 and November 2018 formed an external validation set. The patients were all recruited from inpatient settings. Inclusion criteria included individuals with symptoms of chest pain, cardiovascular risk factors, ischemic changes in electrocardiogram, or elevated myocardial enzymes. Each subject underwent coronary angiography for confirmation of presence and severity of CAD prior to enrollment.

In this study, CAD patients were divided into two groups: stable CADs and AMIs. On the basis of clinical symptoms, the extent of arterial blockage, and the degree of myocardial injury, stable CADs were defined as those patients with angina pectoris including recurrent, transient episodes of chest pain reflecting demand-supply mismatch or < 50% stenosis in all coronary vessels [18]. AMIs referred to the patients with severe and persistent chest pain, typical electrocardiogram changes, and elevated serum biomarkers of myocardial necrosis like cardiac troponins [19]. Patients with any form of malignant tumor, myocardial bridging, percutaneous intervention, and those who underwent a recent surgical procedure and/or had severe heart failure with left ventricular ejection fraction (LVEF) < 20% were excluded. All the participants provided written informed consents. The study was performed in accordance with the Declaration of Helsinki and was approved by all centers.

### 2.2. Data Collection

The data collection for the enrolled patients encompassed questionnaires, data collection forms, and blood samples. Baseline characteristics including age, gender, body mass index (BMI), hypertension history, diabetes history, current smoking status, blood sugar, total cholesterol (TC), triglyceride (TG), high-density lipoprotein cholesterol (HDL-C), low-density lipoprotein cholesterol (LDL-C) and LVEF were collected from the hospital medical record system and inputted into our study database. Data quality checks and controls were conducted to identify and correct errors and deviations in the data in a timely manner. Before coronary angiography, blood was collected for the following laboratory tests.

### 2.3. Sample Preparation

All the plasma samples were collected from the patients prior to initial coronary angiography and were immediately frozen at −80°C before use. To extract the amino acids, 50 *μ*L plasma was thawed at 4°C and mixed with 150 *μ*L methanol (containing isotope-labeled internal standards, 15 ng/mL ^13^C-N-methylproline, 1 *μ*g/mL ^13^C-N,N-dimethylserine, 10 ng/mL ^13^C-N,N-dimethylleucine, 3 *μ*g/mL ^13^C_6_-L-leucine, and 0.5 *μ*g/mL ^13^C-N_5_,N_5_-dimethylornithine) by vortexing for 30 s. Subsequently, the mixture was centrifuged at a speed of 13,000 rpm/min at 4°C for 10 min to precipitate the protein. After that, 150 *μ*L of the supernatants was carefully transferred and dried under a gentle stream of N_2_ at room temperature. Finally, the obtained residues were reconstituted with 100 *μ*L of 50% acetonitrile aqueous solution and 1 *μ*L injected for analysis by mass spectrometry (MS). The pooled quality control (QC) samples were prepared by mixing equal volumes of each plasma and were pretreated under the same conditions as the study samples.

### 2.4. Quantitative Analysis of Amino Acids

In our previous research, we detected 27 amino acids (N,N-dimethylleucine, leucine + isoleucine, phenylalanine, tryptophan, N-methylproline, valine, methionine, N,N-dimethylglycine, proline, tyrosine, alanine, threonine, glycine, glutamic acid, glutamine, serine, asparaginate, citrulline, N6-dimethyllysine, N6-trimethyllysine, 1-methylhistidine, 3-methylhistidine, arginine, histidine, lysine, ornithine, and cystine) in the blood samples of patients with CAD [17]. Targeted analysis of the 27 amino acids was performed on an Agilent 6470 triple quadrupole LC/MS system. An ACQUITY UPLC BEH Amide column (2.1 × 100 mm, 1.7 *μ*m) (Waters, USA) was employed for the separation of the plasma amino acids. The column was operated at 40°C at a flow rate of 0.4 mL/min. Mobile phase A was ultrapure water containing 1 mM ammonium formate, 1 mM ammonium acetate, and 0.2% formic acid. Mobile phase B was 95% aqueous acetonitrile solution containing 0.5 mM ammonium formate, 0.5 mM ammonium acetate, and 0.2% formic acid. The elution program started at an initial gradient of 90% phase B, then decreased to 80% at 1–5 min, decreased to 60% at 5–7 min, changed to 50% at 7–10 min, and immediately back to the initial gradient and kept for 8 min to equilibrate the system. The MS detection was carried out in the positive ion mode, and detailed parameters were set as follows: the drying gas was set at 300°C at a flow rate of 5 L/min; the sheath gas temperature was 250°C, and the sheath gas flow was 11 L/min; the capillary voltage was set at 3500 V, and the nebulizer was 45 psi.

Stable isotope dilution liquid chromatography tandem mass spectrometry (LC-MS/MS) analyses were employed to quantify the amino acids in the plasma. Commercially unavailable ^13^C-labeled internal standards including ^13^C-N-methylproline, ^13^C-N,N-dimethylserine, and ^13^C-N,N-dimethylleucine were synthesized. The details of synthetic routes are shown in the supporting information (available here). To confirm the reliability of the targeted analytical strategy, the method was validated for linearity, lower limit of quantification, accuracy, and precision. Detailed descriptions are provided in the supporting information.

### 2.5. Statistical Analysis

The continuous demographic characteristics were presented as **mean ± standard deviation** (mean ± SD), and categorical variables as percentage (%). All statistical tests were two-sided, and *p* < 0.05 was considered statistically significant unless stated otherwise. All statistical analyses were performed in the software R (version 4.0.3).

### 2.6. Model Development

The prediction model was developed using a combination of clinical variables and amino acid profiles.

The following steps and methodologies were involved. The base prediction model included 12 clinical variables: age, gender, BMI, hypertension history, diabetes history, current smoking status, blood sugar, TC, TG, HDL-C, LDL-C, and LVEF. These variables were selected based on their known association with CAD and AMI.

All amino acid concentrations were first log-transformed prior to modelling to obtain approximately normal distributions. Univariate logistic regression was conducted to screen for statistically significant variates. The amino acid-assisted model incorporated five amino acids identified through univariate logistic regression as significant biomarkers: lysine, methionine, tryptophan, tyrosine, and N6-trimethyllysine. Amino acid concentrations were first log-transformed to achieve approximately normal distributions. Risk scores were calculated by summing the products of each significant amino acid concentration and its corresponding regression coefficient. To account for potential confounding due to different recruitment centers, risk scores were Pareto scaled, which helps standardize the scores and reduce the influence of center-specific variations.

The receiver operating characteristic (ROC) curves were used to evaluate the performance of both the base and amino acid-assisted models. The areas under the curve (AUC) for the discovery and validation phases demonstrated the improved predictive capability of the amino acid-assisted model. A nomogram was constructed to visualize the prediction model, providing a graphical representation of the model that integrates multiple predictors to estimate the probability of AMI. The performance of the models was validated using an independent patient cohort. Calibration curves were generated to assess the agreement between predicted and observed outcomes, ensuring the model's accuracy. The consistency index (C-index) was calculated to further validate the model's performance.

## 3. Results

### 3.1. Clinical Characteristics and Study Design

**Figure 1:**
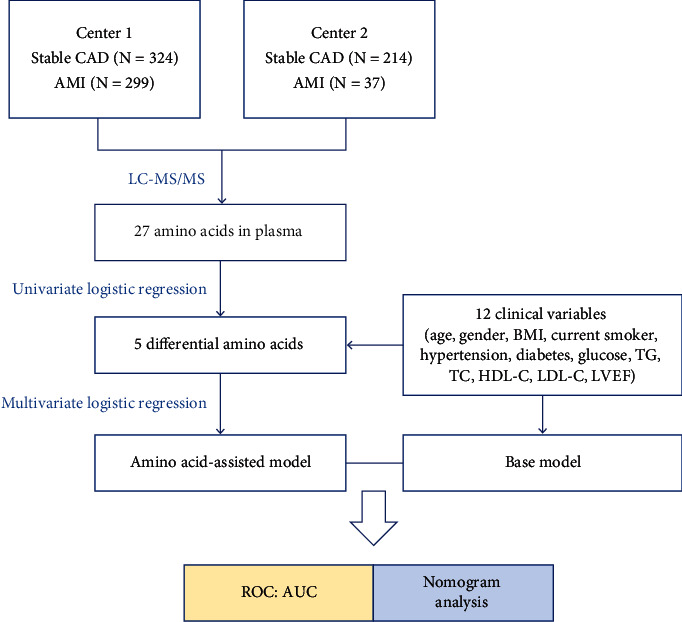
Brief outline of the study design. A total of 874 CAD patients from two independent centers were involved in this work. A targeted metabolomics based on isotope-labeled internal standards for accurate quantification of 27 amino acids (including seven methylated amino acids) using liquid chromatography tandem mass spectrometry (LC-MS/MS) was developed. A comparison of amino acid profiling for stable CAD versus AMI was then performed. Five amino acids were screened as the potential biomarkers by univariate logistic regression (*p* < 0.05). ROC curve and nomogram analyses were employed to evaluate the predictive performances of the base and amino acid-assisted models in the two centers. CAD, coronary artery disease; AMI, acute myocardial infarction; BMI, body mass index; TG, triglyceride; TC, total cholesterol; HDL-C, high-density lipoprotein cholesterol; LDL-C, low-density lipoprotein cholesterol; LVEF, left ventricular ejection fraction; ROC, receiver operating characteristic.

A total of 874 patients with stable CAD or AMI were enrolled in this study. Center 1 (*n* = 623) comprising 324 stable CADs and 299 AMI subjects was employed as the discovery phase, and Center 2 (*n* = 251), which was composed of 214 stable CADs and 37 AMI participants, was used for external validation. The enrollment process is shown in Figure S1. The baseline characteristics of the participants are shown in Table 1. The study design is outlined in Figure 1. First, a targeted strategy was developed to accurately quantify 27 amino acids. Then, univariate logistic regression was used to screen for the differential amino acids from the comparison of stable CAD versus AMI. Finally, ROC curve and nomogram analyses were employed to evaluate the prediction performances of the base and amino acid-assisted models.

### 3.2. Quantification of Plasma Amino Acids in CAD Patients

Accurate targeted quantification of endogenous metabolites is largely dependent on stable isotope internal standards. Three commercially unavailable ^13^C-labeled methylated amino acids, including ^13^C-N-methylproline, ^13^C-N,N-dimethylserine, and ^13^C-N,N-dimethylleucine, were synthesized via reductive alkylation using ^13^C-labeled formaldehyde under a palladium-hydrogen system (Figure S2). The synthesized methylated products were characterized by high-resolution mass spectrometric analyses and nuclear magnetic resonance (NMR) as shown in Figure S3. Multiple reaction monitoring was employed to accurately capture the amino acids. Most amino acids produced abundant fragment ion of [M + H-46 (H_2_O + CO)]^+^ under positive mode—the detailed ion transitions monitored are provided in Table S1. A representative total ion chromatogram of the 27 amino acids generated from the LC-MS/MS analysis is presented in Figure S4. Most of the amino acids produced adequate retentions, good peak shapes, and stable MS signals within 10 min. Leucine and isoleucine could not achieve baseline separation since they have the same mass and exhibit similar chromatographic behavior. Hence, they were combined as leucine + isoleucine for analysis. A stable isotope-labeled internal standard calibration strategy was applied for the accurate quantification of the 27 amino acids. Details of the results including linearity, lower limit of quantitation, accuracy, and precision are summarized in Tables S2–S3.

### 3.3. Clinical ROC Evaluation

**Figure 2:**
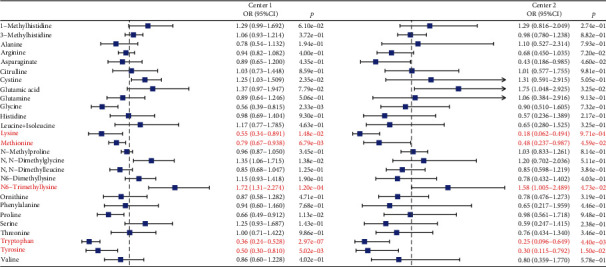
Univariate logistic regression analysis of 27 amino acids in Center 1 and Center 2. Five amino acids with *p* < 0.05 from the comparison of stable CAD (coronary artery disease) versus AMI (acute myocardial infarction) in both Center 1 and Center 2 were identified as potential biomarkers. OR, odds ratio.

Univariate logistic regression was employed to identify the differential amino acids between stable CADs and AMIs. Nine amino acids with *p* < 0.05, thus, glycine, lysine, methionine, proline, tryptophan, tyrosine, cystine, N6-trimethyllysine, and N,N-dimethylglycine, were identified to be associated with CAD progression in the discovery phase (Figure 2). Notably, changes in the levels of five amino acids including decreased lysine, methionine, tryptophan, tyrosine, and increased N6-trimethyllysine were replicated in the external validation set (Figure 2). A panel consisting of these five differential amino acids was therefore selected for risk score calculation for each person (Figure 2). The visualized scatterplot showed that AMI patients had higher risk scores and were visibly distinguished from the stable CAD group (Figure S5). In addition, the relationship between CAD progression and risk score was assessed by a restricted cubic spline model. Spline plots revealed a positive correlation between risk score value and CAD progression, indicating that the amino acid plasma concentrations were highly correlated with the risk of AMI (Figure S6).

**Figure 3:**
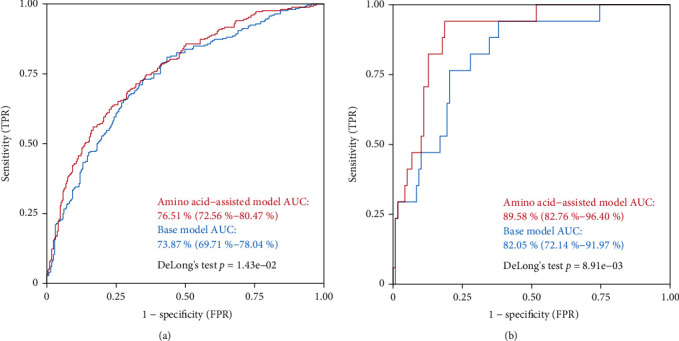
ROC curve analyses of the base model (Model 1) and amino acid-assisted model (Model 2) for stable CAD versus AMI in (a) Center 1 and (b) Center 2. ROC, receiver operating characteristic; CAD, coronary artery disease; AMI, acute myocardial infarction; AUC, areas under the curve.

Using the clinical variables (age, gender, BMI, current smoking status, history of hypertension, history of diabetes, blood glucose, TG, TC, HDL-C, LDL-C, and LVEF), a base prediction model by the logistic regression was developed to distinguish AMI from stable CAD. An amino acid-assisted prediction model, combining the five amino acid biomarkers with clinical variables, was established to enhance risk prediction of AMI in stable CAD. The clinical diagnoses of the two models were evaluated by ROC curve analysis in the discovery phase. Compared with the base model, the amino acid-assisted model showed significantly enhanced diagnostic performance for the comparison of stable CAD versus AMI. The AUC increased from 73.87% (95% CI: 69.71%–78.04%) to 76.51% (95% CI: 72.56%–80.47%, Delong's test *p* = 1.43e‐02) (Figure 3(a)). It is worth noting that the external validation set further confirmed this finding. Hence, the AUC significantly improved from 82.05% (95% CI: 72.14%–91.97%) to 89.58% (95% CI: 82.76%–96.40%, Delong's test *p* = 8.91e‐03) (Figure 3(b)).

### 3.4. Development of Nomogram

**Figure 4:**
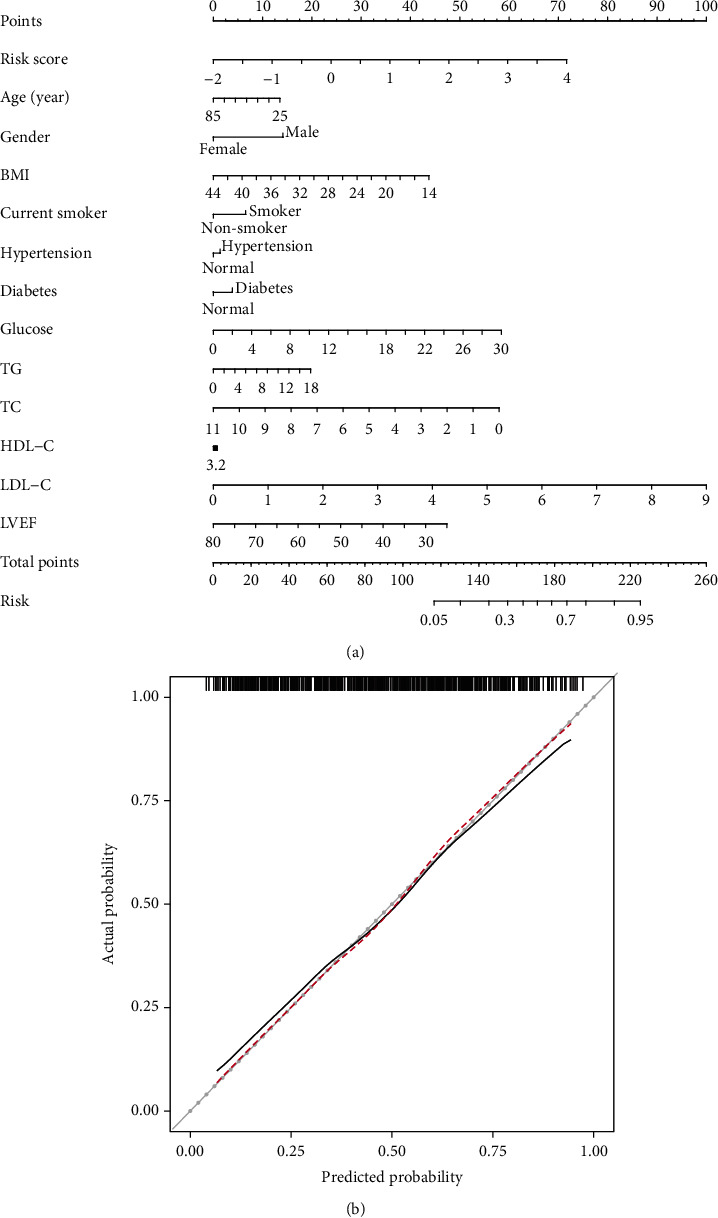
Amino acid-assisted nomogram for predicting (a) AMI risk and (b) calibration curve analysis. The calibration curve of the nomogram was conducted to evaluate the agreement between the actual observation and the predicted probability of AMI. AMI, acute myocardial infarction; BMI, body mass index; TG, triglyceride; TC, total cholesterol; HDL-C, high-density lipoprotein cholesterol; LDL-C, low-density lipoprotein cholesterol; LVEF, left ventricular ejection fraction.

The nomogram prediction model for AMI risk in Center 1 (discovery phase) is illustrated in Figure 4(a). The risk score calculated on the basis of the five differential amino acids showed a significant effect on AMI risk prediction. The predictive performance of the nomogram model was evaluated using C-index and calibration curve analysis. In the base model, the C-index was 73.87% (95% CI: 69.71%–78.04%) for stable CAD versus AMI (Table S4). Notably, the C-index of the amino acid-assisted model was improved, reaching up to 76.51% (95% CI: 72.56%–80.47%) (Table S4). Furthermore, the calibration curve of the nomogram model demonstrated good agreement between the actual observation and predicted probability for AMI (Figure 4(b)).

## 4. Discussion

In this study, we analyzed 27 amino acids in the plasma of 874 CAD patients and identified five amino acids as potential biomarkers for AMI prediction. Leveraging the five amino acids alongside 12 clinical parameters, we crafted a promising predictive model for the early detection of AMI among CAD. Notably, we found that the five amino acids significantly bolstered the model's discrimination power, leading to a substantial enhancement in its predictive capabilities.

The development and progression of CAD are multifaceted, encompassing factors such as gender, age, exposure to adverse environmental conditions, genomic variations, and metabolome alterations. Prior research has demonstrated that clinical risk models, augmented by biomarkers, outperform their standalone counterparts in predicting CAD outcomes [20–22]. In this work, five amino acids (lysine, methionine, tryptophan, tyrosine, and N6-trimethyllysine) verified through two distinct datasets significantly increased the predictive abilities of the models for the early prediction of AMI among CAD patients.

Methionine serves as a precursor in homocysteine metabolism. Homocysteine is a well-known risk factor of CAD. Studies have shown that CAD patients with elevated homocysteine levels tend to have a higher incidence of AMI [23]. However, to our knowledge, no direct link between methionine levels and AMI risk has been reported. Carnitine, a downstream metabolite of methionine, plays a pivotal role in the transportation of long-chain fatty acids into the mitochondrial matrix and offers cardioprotection by reducing oxidative stress, inflammation, and necrosis of cardiomyocytes [24]. L-Carnitine deficiency has been associated with AMI [25]. In our research, we observed a notable decrease in methionine level among AMI patients compared to those with stable CAD patients. We hypothesize that the low methionine level may stem from the depletion of carnitine during AMI. Interestingly, a previous cohort study concurred with our findings, revealing that low plasma methionine was associated with an increased risk of AMI among patients with elevated levels of atherogenic lipids [26].

The relationship between tryptophan and CAD has been widely investigated. Decreased tryptophan levels have emerged as a predictive marker for adverse cardiac events [27]. Furthermore, downstream tryptophan metabolic intermediates, such as kynurenine and kynurenic acid, have been identified as biomarkers for AMI. Increased tryptophan degradation via the kynurenine pathway was found to be strongly correlated with the risk of AMI among patients with suspected stable angina pectoris [28].

N6-Trimethyllysine, a precursor of TMAO, is a well-recognized risk factor for cardiovascular events. Prospective cohort studies have established a clear link between N6-trimethyllysine and the progression of CAD as well as the prognosis of AMI [13, 29]. In line with these findings, the findings of our study also indicate that an increased risk of AMI is closely tied to elevated levels of circulating N6-trimethyllysine. While the precise mechanisms underlying this robust association between N6-trimethyllysine and AMI remain elusive, this marker holds promising potential in predicting the risk of AMI.

Lysine and tyrosine, both fundamental amino acids, occupy pivotal roles in sustaining human physiological functions. Our research sheds light on a negative correlation between these amino acids and the risk of AMI. Nevertheless, the direct link between lysine and tyrosine with AMI remains shrouded in scientific uncertainty. As a free molecule, lysine takes part in the antioxidant response and engages in protein modifications [30]. Notably, a study revealed that a higher intake of lysine inversely correlates with the risk of nonalcoholic fatty liver disease [31]. It may assist in safeguarding myocardial cells by mitigating oxidative stress and inflammatory responses following AMI. Additionally, tyrosine serves as a precursor for neurotransmitters such as dopamine and norepinephrine, which are integral to cardiovascular regulation [32]. Increased tyrosine intake has been shown to exert beneficial effects on psychological functioning [33]. Given that AMI can disrupt the delicate balance of the neurotransmitter system, tyrosine supplementation may aid in restoring this balance. From a broader perspective, considering their indispensability in protein synthesis, lysine and tyrosine are poised to contribute significantly to the recovery process following a myocardial infarction.

There are indeed several limitations in this study. Firstly, the intricate relationship between plasma amino acid concentrations and the risk of AMI remains elusive due to the absence of prospective data. To address this, future studies should consider larger, population-based prospective cohorts to validate our findings. Secondly, further in vitro/vivo biological studies are imperative to definitively confirm the pivotal roles of amino acids, particularly methylated amino acids, in the progression of CAD. Finally, the current model exhibited a moderate AUC, suggesting that its predictive performance has the potential for significant enhancement. To enhance its predictive power, future research could incorporate additional clinical indices into a more comprehensive model.

## 5. Conclusions

Significant associations have been observed between amino acid levels and the progression of CAD. The five amino acid-assisted model remarkably improved the risk prediction of AMI in patients with stable CAD. Consequently, this advancement allows for the timely implementation of secondary prevention strategies to facilitate early intervention.

## Figures and Tables

**Table 1:** Baseline characteristics of 874 CAD patients.

	**Center 1**			**Center 2**		
	**Stable CAD**	**AMI**	**p** ** value**	**Stable CAD**	**AMI**	**p** ** value**
	**(** **N** = 324**)**	**(** **N** = 299**)**		**(** **N** = 214**)**	**(** **N** = 37**)**	
Age (year)	64.42 ± 9.04	64.06 ± 11.78	6.74e‐01^∗^	60.38 ± 11.87	60.51 ± 9.04	9.58e‐01^∗^
Gender (male, %)	51.54	75.84	5.91**e**‐10^∗∗^	56.81	72.97	9.58e‐02^∗∗^
BMI	25.32 ± 3.54	24.45 ± 3.53	2.89**e**‐03^∗^	25.14 ± 3.62	25.46 ± 3.19	5.92e‐01^∗^
Current smoker (%)	25.93	44.48	1.82**e**‐06^∗∗^	25.47	32.43	4.94e‐01^∗∗^
Comorbidity						
Hypertension (%)	65.12	62.08	4.81e‐01^∗∗^	61.5	59.46	9.58e‐01^∗∗^
Diabetes (%)	19.14	22.41	3.64e‐01^∗∗^	16.9	29.73	1.06e‐01^∗∗^
Laboratory data						
Glucose (mmol/L)	6.32 ± 2.24	7.38 ± 3.54	1.18**e**‐05^∗^	5.9 ± 2.28	6.89 ± 3.06	1.67e‐01^∗^
TG (mmol/L)	1.9 ± 1.48	1.93 ± 1.9	8.07e‐01^∗^	1.72 ± 1.12	2.21 ± 1.88	1.77e‐01^∗^
TC (mmol/L)	4.4 ± 1.09	4.52 ± 1.21	2.28e‐01^∗^	4.29 ± 1.01	4.56 ± 1.01	1.83e‐01^∗^
HDL-C (mmol/L)	1.14 ± 0.3	1.07 ± 0.29	4.67**e**‐03^∗^	1.24 ± 0.43	1.07 ± 0.32	1.17**e**‐02^∗^
LDL-C (mmol/L)	2.67 ± 0.91	2.89 ± 1.01	6.17**e**‐03^∗^	2.68 ± 0.91	2.89 ± 1.03	3.06e‐01^∗^
Creatinine (*μ*mol/L)	71.59 ± 18.62	83.33 ± 34.32	1.25**e**‐07^∗^	71.78 ± 34.25	74.63 ± 24.75	6.53e‐01^∗^
BUN (mmol/L)	6.20 ± 2.51	6.29 ± 2.74	7.39e‐01^∗^	5.61 ± 1.68	6.52 ± 3.28	1.65**e**‐02^∗^
LVEF (%)	61.61 ± 8.48	57.62 ± 8.66	3.11**e**‐08^∗^	61.3 ± 7.06	57.31 ± 7.69	1.29**e**‐02^∗^

*Note:* Values are mean ± SD or %.

Abbreviations: AMI, acute myocardial infarction; BMI, body mass index; CAD, coronary artery disease; HDL-C, high-density lipoprotein cholesterol; LDL-C, low-density lipoprotein cholesterol; LVEF, left ventricular ejection fraction; TC, total cholesterol; TG, triglyceride.

The bold *p* value indicates *p* < 0.05.

^*^ *p* values were calculated by Student's *t*-test for continuous characteristics.

^**^ *p* values were calculated by the Chi-square test for binary characteristics.

## Data Availability

We have provided the data in the supporting information file.

## Supporting Information

Supporting Information Additional supporting information can be found online in the Supporting Information section. Supporting methods: the synthesis and characterization of ^13^C-labeled internal standards, method validation. Supporting figures—Figure S1: the enrollment process for clinical cases; Figure S2: the synthetic route of ^13^C-labeled internal standards; Figure S3: the nuclear magnetic resonance (NMR) analyses for the synthesized ^13^C-labeled reagents; Figure S4: representative total ion chromatogram of the 27 amino acids generated from the LC-MS/MS analysis; Figure S5: scatterplot of calculated risk score of each participant in Center 1 and Center 2; Figure S6: restricted cubic spline analysis of the relationship between coronary artery disease progression and risk score in Center 1 and Center 2. Supporting tables—Table S1: the MS parameters of the 27 amino acids and five ^13^C-labeled internal standards; Table S2: the linearity, lower limit of quantitation (LLOQ), and accuracy of the LC-MS/MS method; Table S3: inter- and intraday precision of the analytical method; Table S4: the C-indices of base model and amino acid-assisted model.

## References

[1] Yeh R. W., Sidney S., Chandra M., Sorel M., Selby J. V., Go A. S. (2010). Population trends in the incidence and outcomes of acute myocardial infarction. *New England Journal of Medicine*.

[2] Nichols M., Townsend N., Scarborough P., Rayner M. (2014). Cardiovascular disease in Europe 2014: epidemiological update. *European Heart Journal*.

[3] Reed G. W., Rossi J. E., Cannon C. P. (2017). Acute myocardial infarction. *The Lancet*.

[4] Shibata T., Kawakami S., Noguchi T. (2015). Prevalence, clinical features, and prognosis of acute myocardial infarction attributable to coronary artery embolism. *Circulation*.

[5] Thygesen K., Alpert J. S., Jaffe A. S. (2012). Third universal definition of myocardial infarction. *Journal of the American College of Cardiology*.

[6] Braunwald E. (2012). Unstable angina and non-ST elevation myocardial infarction. *American Journal of Respiratory and Critical Care Medicine*.

[7] Du X., You H., Li Y. (2018). Relationships between circulating branched chain amino acid concentrations and risk of adverse cardiovascular events in patients with STEMI treated with PCI. *Scientific Reports*.

[8] Tobias D. K., Lawler P. R., Harada P. H. (2018). Circulating branched-chain amino acids and incident cardiovascular disease in a prospective cohort of US women. *Circulation: Genomic and Precision Medicine*.

[9] Bønaa K. H., Njølstad I., Ueland P. M. (2006). Homocysteine lowering and cardiovascular events after acute myocardial infarction. *New England Journal of Medicine*.

[10] Pedersen E. R., Svingen G. F. T., Schartum-Hansen H. (2013). Urinary excretion of kynurenine and tryptophan, cardiovascular events, and mortality after elective coronary angiography. *European Heart Journal*.

[11] Eussen S. J. P. M., Ueland P. M., Vollset S. E. (2015). Kynurenines as predictors of acute coronary events in the Hordaland Health Study. *International Journal of Cardiology*.

[12] Sreekumar A., Poisson L. M., Rajendiran T. M. (2009). Metabolomic profiles delineate potential role for sarcosine in prostate cancer progression. *Nature*.

[13] Li X. S., Obeid S., Wang Z. (2019). Trimethyllysine, a trimethylamine N-oxide precursor, provides near- and long-term prognostic value in patients presenting with acute coronary syndromes. *European Heart Journal*.

[14] Dunn W. B., Broadhurst D., Begley P. (2011). Procedures for large-scale metabolic profiling of serum and plasma using gas chromatography and liquid chromatography coupled to mass spectrometry. *Nature Protocols*.

[15] Gouveia M. J., Brindley P. J., Santos L. L., Correia da Costa J. M., Gomes P., Vale N. (2013). Mass spectrometry techniques in the survey of steroid metabolites as potential disease biomarkers: a review. *Metabolism*.

[16] Cheng M. L., Wang C. H., Shiao M. S. (2015). Metabolic disturbances identified in plasma are associated with outcomes in patients with heart failure: diagnostic and prognostic value of metabolomics. *Journal of the American College of Cardiology*.

[17] Fan Y., Li Y., Chen Y. (2016). Comprehensive metabolomic characterization of coronary artery diseases. *Journal of the American College of Cardiology*.

[18] Ford T. J., Corcoran D., Berry C. (2018). Stable coronary syndromes: pathophysiology, diagnostic advances and therapeutic need. *Heart*.

[19] Thygesen K., Alpert J. S., Jaffe A. S. (2018). Fourth universal definition of myocardial infarction (2018). *Journal of the American College of Cardiology*.

[20] de Filippi C. R., de Lemos J. A., Christenson R. H. (2010). Association of serial measures of cardiac troponin T using a sensitive assay with incident heart failure and cardiovascular mortality in older adults. *JAMA*.

[21] Schnabel R. B., Schulz A., Messow C. M. (2010). Multiple marker approach to risk stratification in patients with stable coronary artery disease. *European Heart Journal*.

[22] Velagaleti R. S., Gona P., Larson M. G. (2010). Multimarker approach for the prediction of heart failure incidence in the community. *Circulation*.

[23] Al-Obaidi M. K., Stubbs P. J., Collinson P., Conroy R., Graham I., Noble M. I. M. (2000). Elevated homocysteine levels are associated with increased ischemic myocardial injury in acute coronary syndromes. *Journal of the American College of Cardiology*.

[24] Wang Z. Y., Liu Y. Y., Liu G. H., Lu H. B., Mao C. Y. (2018). L-Carnitine and heart disease. *Life Sciences*.

[25] Spagnoli L. G., Corsi M., Villaschi S., Palmieri G., Maccari F. (1982). Myocardial carnitine deficiency in acute myocardial infarction. *The Lancet*.

[26] Dhar I., Lysne V., Seifert R., Svingen G. F. T., Ueland P. M., Nygård O. K. (2018). Plasma methionine and risk of acute myocardial infarction: effect modification by established risk factors. *Atherosclerosis*.

[27] Yu E., Ruiz-Canela M., Guasch-Ferré M. (2017). Increases in plasma tryptophan are inversely associated with incident cardiovascular disease in the prevención con dieta mediterránea (PREDIMED) study. *The Journal of Nutrition*.

[28] Pedersen E. R., Tuseth N., Eussen S. J. P. M. (2015). Associations of plasma kynurenines with risk of acute myocardial infarction in patients with stable angina pectoris. *Arteriosclerosis, Thrombosis, and Vascular Biology*.

[29] Bjørnestad E. Ø., Olset H., Dhar I. (2020). Circulating trimethyllysine and risk of acute myocardial infarction in patients with suspected stable coronary heart disease. *Journal of Internal Medicine*.

[30] Tan Y., Chrysopoulou M., Rinschen M. M. (2023). Integrative physiology of lysine metabolites. *Physiological Genomics*.

[31] Li X., Ma W., Yang T. (2024). Higher intakes of lysine, threonine and valine are inversely associated with non-alcoholic fatty liver disease risk: a community-based case-control study in the Chinese elderly. *Food Science and Human Wellness*.

[32] Coull N., Chrismas B., Watson P., Horsfall R., Taylor L. (2016). Tyrosine ingestion and its effects on cognitive and physical performance in the heat. *Medicine & Science in Sports & Exercise*.

[33] Hase A., Jung S. E., aan het Rot M. (2015). Behavioral and cognitive effects of tyrosine intake in healthy human adults. *Pharmacology Biochemistry and Behavior*.

