# Supplementary material for: Improved Risk Prediction of Acute Myocardial Infarction in Patients With Stable Coronary Artery Disease Using an Amino Acid-Assisted Model
Source: Cardiovasc Ther. 2024 Aug 30;2024:9935805. doi: 10.1155/2024/9935805 (PMC11379511; doi:10.1155/2024/9935805)
Supplement: Supporting Information — Additional supporting information can be found online in the Supporting Information section. Supporting methods: the synthesis and characterization of 13C-labeled internal standards, method validation. Supporting figures—Figure S1: the enrollment process for clinical cases; Figure S2: the synthetic route of 13C-labeled internal standards; Figure S3: the nuclear magnetic resonance (NMR) analyses for the synthesized 13C-labeled reagents; Figure S4: representative total ion chromatogram of the 27 amino acids generated from the LC-MS/MS analysis; Figure S5: scatterplot of calculated risk score of each participant in Center 1 and Center 2; Figure S6: restricted cubic spline analysis of the relationship between coronary artery disease progression and risk score in Center 1 and Center 2. Supporting tables—Table S1: the MS parameters of the 27 amino acids and five 13C-labeled internal standards; Table S2: the linearity, lower limit of quantitation (LLOQ), and accuracy of the LC-MS/MS method; Table S3: inter- and intraday precision of the analytical method; Table S4: the C-indices of base model and amino acid-assisted model. [file 9935805.f1.docx]

**Improved Risk Prediction of Acute Myocardial Infarction in Patients with Stable Coronary Artery Disease Using an Amino Acid-Assisted Model**

Yi-Jing Zhao^1†^, Feng-Xiang Wang^2†^, Hao Lv^1^, Yaoyao Qu^1^, Lian-Wen Qi^1^*, Yong Li^3^*, Pingxi Xiao^4^*

^1^State Key Laboratory of Natural Medicines, School of Traditional Chinese Pharmacy, China Pharmaceutical University, Nanjing, China

^2^The Clinical Metabolomics Center, China Pharmaceutical University, Nanjing, China

^3^Department of Cardiology, the Affiliated Wujin Hospital of Jiangsu University, Changzhou, China

^4^Department of Cardiology, The Fourth Affiliated Hospital of Nanjing Medical University, Nanjing, China

*** Correspondence:**

Pingxi Xiao, Department of Cardiology, The Sir Run Run Hospital, Nanjing Medical University, Nanjing, China, email: [xpx@njmu.edu.cn](mailto:xpx@njmu.edu.cn); Yong Li, Department of Cardiology, the Affiliated Wujin Hospital of Jiangsu University, Changzhou, China, email: [13915008198@126.com](mailto:13915008198@126.com); Lian-Wen Qi, State Key Laboratory of Natural Medicines, School of Traditional Chinese Pharmacy, China Pharmaceutical University, Nanjing, China, email: [qilw@cpu.edu.cn](mailto:qilw@cpu.edu.cn).

^†^These authors contributed equally to this work

**Running title:** Amino acid-based prediction of AMI in CAD patients

**Keywords:** Amino acid; Methylated amino acid; Risk prediction; Acute myocardial infarction; Coronary artery disease

# Supplementary Methods:

**The synthesis and characterization of ^13^C**-**labeled internal standards**

The ^13^C-labeled methylated amino acids were synthesized via reductive alkylation. Briefly, 0.4 mmol amino acid, 37.2 mg ^13^C-labeled formaldehyde (1.2 mmol) and 10 mg Pd/C were first added to 5.0 mL of methanol. Then, the mixture was placed under H_2_ atmosphere and vigorously stirred at room temperature under normal pressure for 24 h. The reaction mixture was subsequently filtered through a Celite pad, concentrated, and dissolved with 1.0 mL of methanol. After that, 30 mL of ether was added to form the crystalline precipitate. The crystals were washed with ether and the crystallization was repeated. Finally, the dried crystals were prepared using the freeze-drying method. The total yields were 44.2% for 13C-N-methylproline, 74.1% for 13C-N,N-dimethylserine and 46.6% for 13C-N,N-dimethylleucine.

The synthetic ^13^C-labeled methylated amino acids were characterized by nuclear magnetic resonance (NMR) and high-resolution mass spectrometric (HRMS) analyses. All the ^1^H NMR and ^13^C NMR spectra were recorded using Bruker spectrometers. Chemical shifts listed in ppm were referenced to (CH_3_)_2_SO (^1^H = 2.5 ppm, ^13^C = 39.6 ppm) in (CD_3_)_2_SO. Data for ^1^H NMR are reported as follows: chemical shifts (δ ppm), multiplicity (s = singlet, d = doublet, m = multiplet), coupling constant (Hz) and integration. Data for ^13^C NMR are reported in terms of chemical shift. High-resolution mass spectra acquisition was performed on a liquid chromatography-quadrupole time-of-flight mass spectrometry (Agilent 6545 Q/TOF-MS, USA).

^13^C-N-methylproline: **^1^H NMR** (300 MHz, DMSO-d6) δ3.37-3.49 (m, 2H), 2.92 (s, 1.5H), 2.79-2.89 (m, 1H), 2.46 (s, 1.5H), 2.14-2.27 (m,1H), 1.85-1.98 (m, 2H), 1.65-1.80 (m, 1H); **^13^C NMR** (75 MHz, DMSO-d6) δ 169.6, 70.3, 55.8, 40.9, 29.1, 23.5; **HRMS** (ESI) calculated for C_5_^13^CH_11_NO_2_ [M+H]^+^ *m/z* 131.0902, found 131.0898.

^13^C-N,N-dimethylserine: **^1^H NMR** (300 MHz, DMSO-d6) δ3.85-3.91 (m, 1H), δ3.73-3.79 (m, 1H), δ3.31-3.37 (m, 1H), 2.91 (d, *J* = 3Hz, 3H), 2.45 (d, *J* = 4.2Hz, 3H); **^13^C NMR** (75 MHz, DMSO-d6) δ 168.1, 70.7, 59.2, 41.9, 41.9; **HRMS** (ESI) calculated for C_3_^13^C_2_H_11_NO_3_ [M+H]^+^ *m/z* 136.0884, found 136.0870.

^13^C-N,N-dimethylleucine: **^1^H NMR** (300 MHz, DMSO-d6) δ3.10-3.17 (m, 1H), 2.64 (d, *J* = 3Hz, 3H), 2.18-2.19 (m, 3H), 1.64-1.77 (m, 1H), 1.38-1.56 (m, 2H), 0.89-0.93 (m, 6H); **^13^C NMR** (75 MHz, DMSO-d6) δ 172.2, 66.2, 40.9, 40.9, 37.7, 24.6, 22.6, 22.3; **HRMS** (ESI) calculated for C_6_^13^C_2_H_17_NO_2_ [M+H]^+^ *m/z* 162.1405, found 162.1415.

**Method validation**

The method performance was evaluated for linearity, lower limit of quantification (LLOQ), accuracy and precision. The linearity was investigated over a wide concentration range (from LLOQ up to 900 μg/mL). All the calibration curves showed good linearity with regression coefficients *r*^2^> 0.99. The lower limit of quantification (LLOQ) was determined as the lowest concentration of the linear range. The accuracy at low, moderate and high levels evaluated as recoveries ranged from 85.5% to 120.4%. The precision was measured at three levels of concentration, thus, low, moderate and high concentrations. The inter-day precision (one replicate of QC sample analyzed on each of three days) calculated by relative standard deviation (RSD%) was less than 6.81% and intra-day precision (three replicates analyzed on same day) was less than 7.40%.

# Supplementary Figure legends:

**Supplementary Figure 1**

The enrollment process for clinical cases.

**Supplementary Figure 2**

The synthetic route of ^13^C-labeled internal standards ^13^C-N-methylproline (A), ^13^C-N,N-dimethylserine (B) and ^13^C-N,N-dimethylleucine (C).

**Supplementary Figure 3**

The nuclear magnetic resonance (NMR) analyses for the synthesized ^13^C-labeled reagents. ^1^H NMR for ^13^C-N-methylproline (A); ^13^C NMR for ^13^C-N-methylproline (B); ^1^H NMR for ^13^C-N,N-dimethylserine (C); ^13^C NMR for ^13^C-N,N-dimethylserine (D); ^1^H NMR for ^13^C-N,N-dimethylleucine (E); ^13^C NMR for ^13^C-N,N-dimethylleucine (F).

**Supplementary Figure 4**

Representative total ion chromatogram of the 27 amino acids generated from the LC-MS/MS analysis. 1. N,N-dimethylleucine; 2. Leucine + Isoleucine; 3. Phenylalanine; 4. Tryptophan; 5. N-methylproline; 6. Methionine; 7. Valine; 8. N,N-dimethylglycine; 9. Proline; 10. Tyrosine; 11. Alanine; 12. Threonine; 13. Glutamic acid; 14. Glycine; 15. Serine; 16. Glutamine; 17. Asparaginate; 18. Citrulline; 19. N6-dimethyllysine; 20. N6-trimethyllysine; 21. 1-methylhistidine; 22. 3-methylhistidine; 23. Arginine; 24. Histidine; 25. Lysine; 26. Ornithine; 27. Cystine.

**Supplementary Figure 5**

Scatterplot of calculated risk score of each participant in Center 1 (A) and Center 2 (B). Blue points refer to patients with stable coronary artery disease and orange points represent the individuals with acute myocardial infarction.

**Supplementary Figure 6**

Restricted cubic spline analysis of the relationship between coronary artery disease progression and risk score in Center 1 (A) and Center 2 (B). OR, odds ratio.

### Supplementary Figure 1.


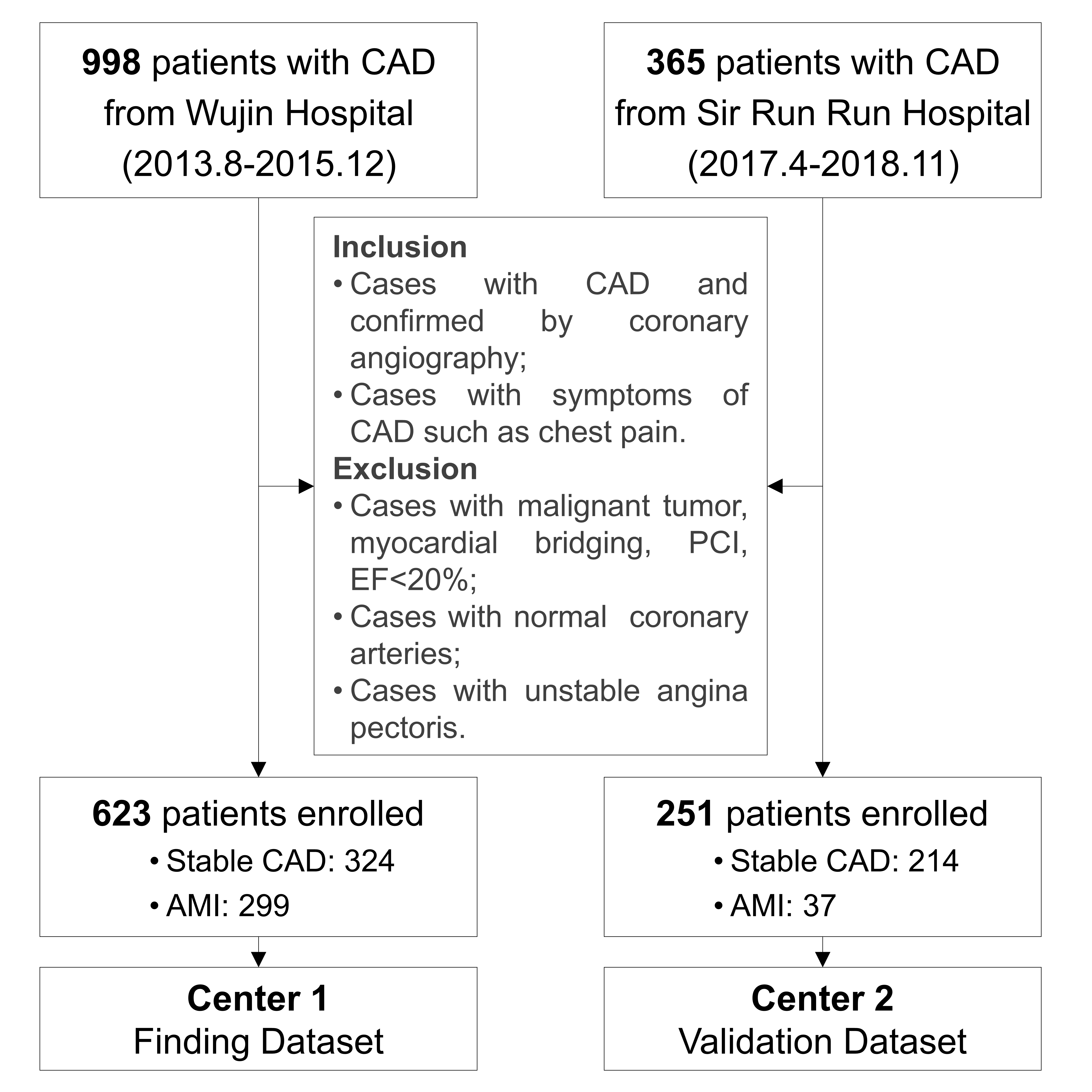


### Supplementary Figure 2.

###

### Supplementary Figure 3.

**
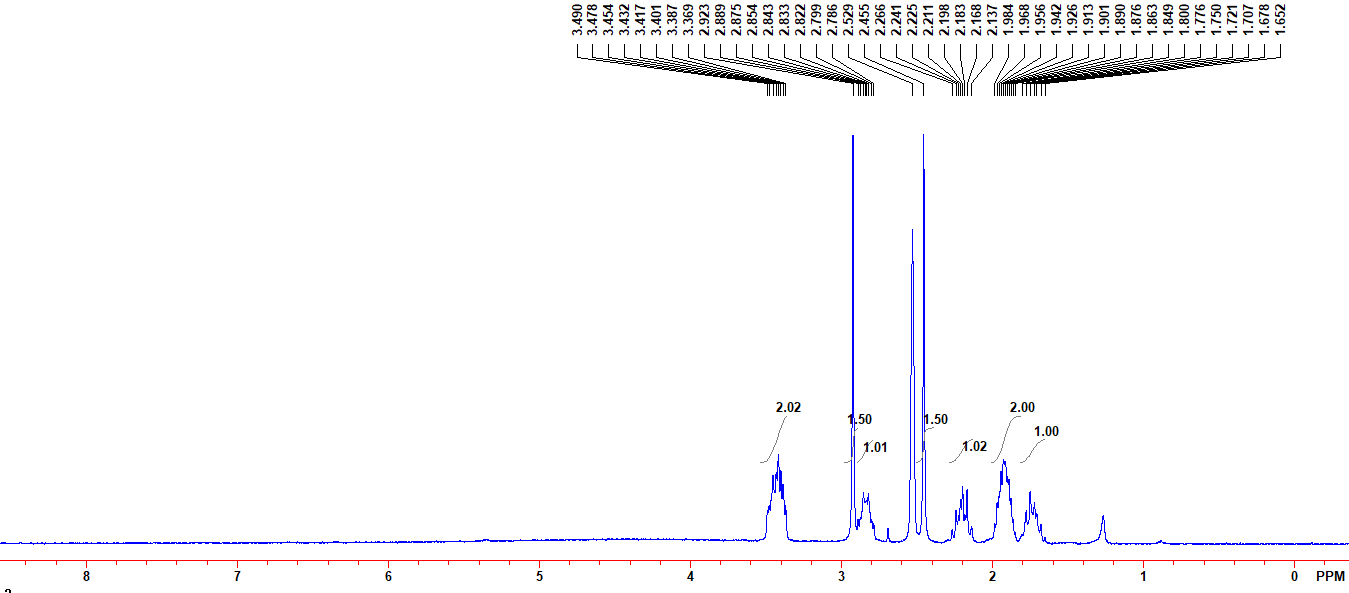
A**

**
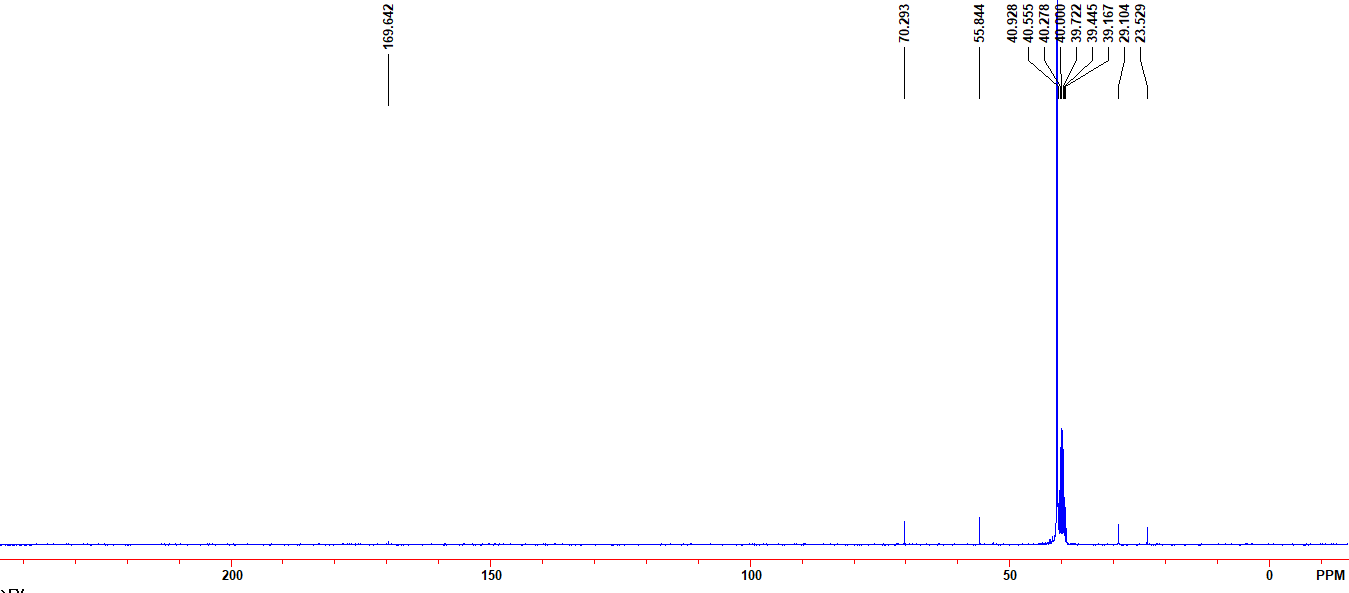
B**

**
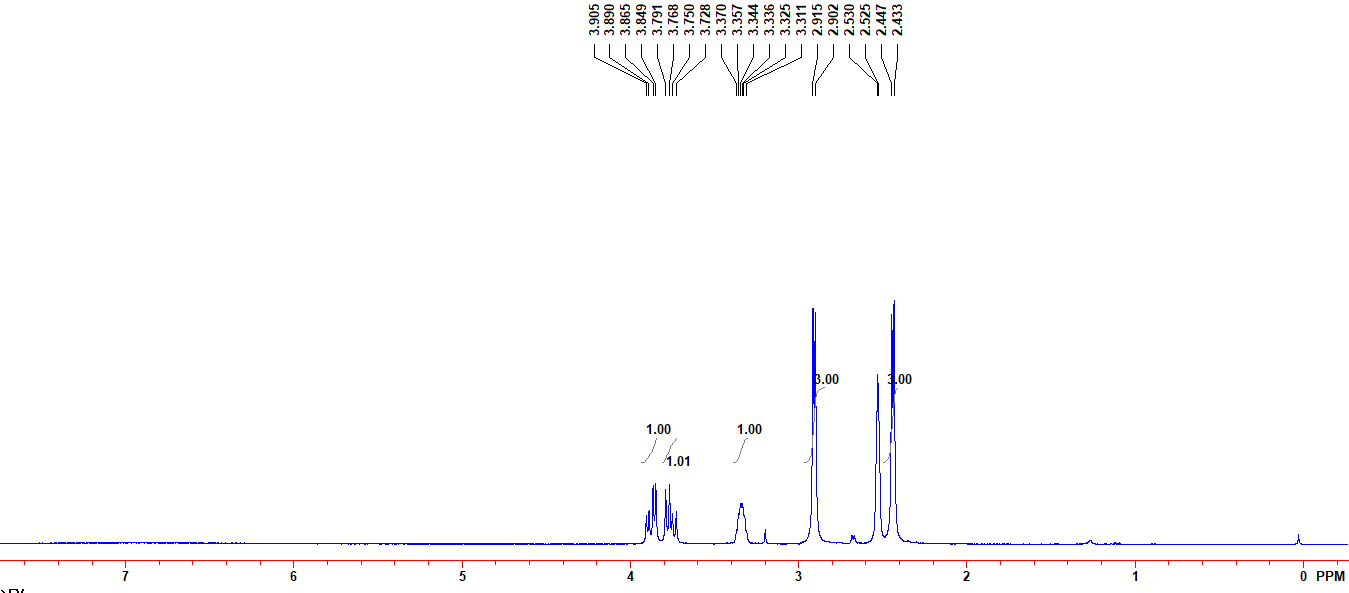
C**

**D**


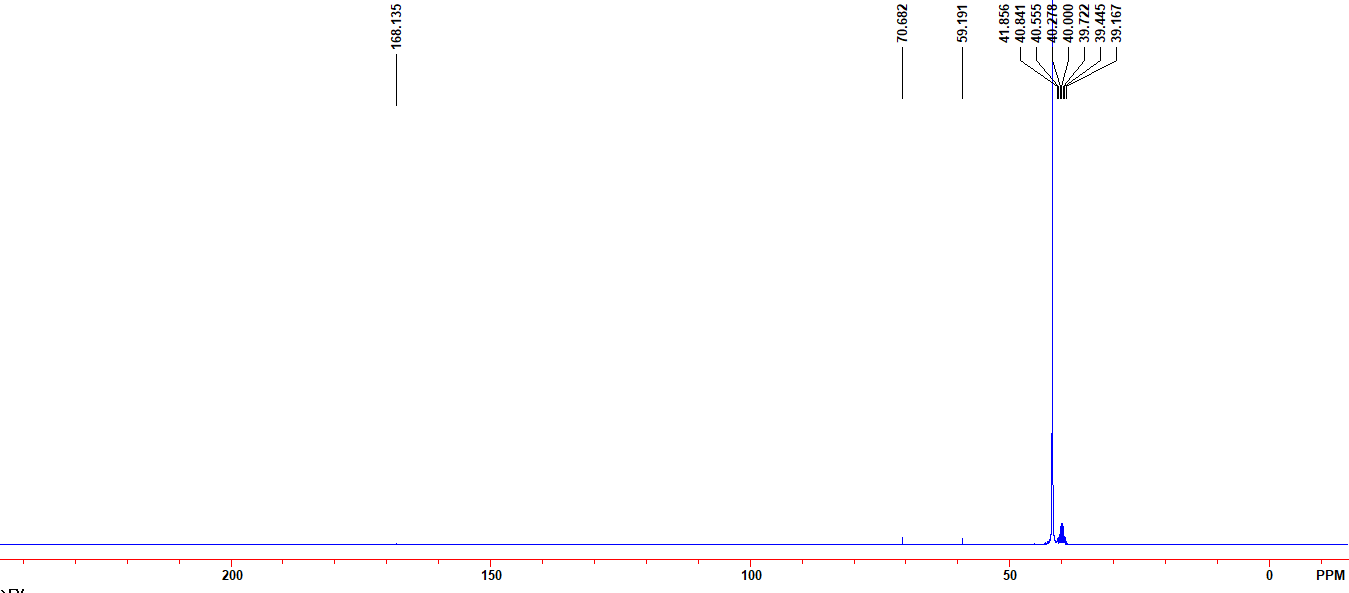

**
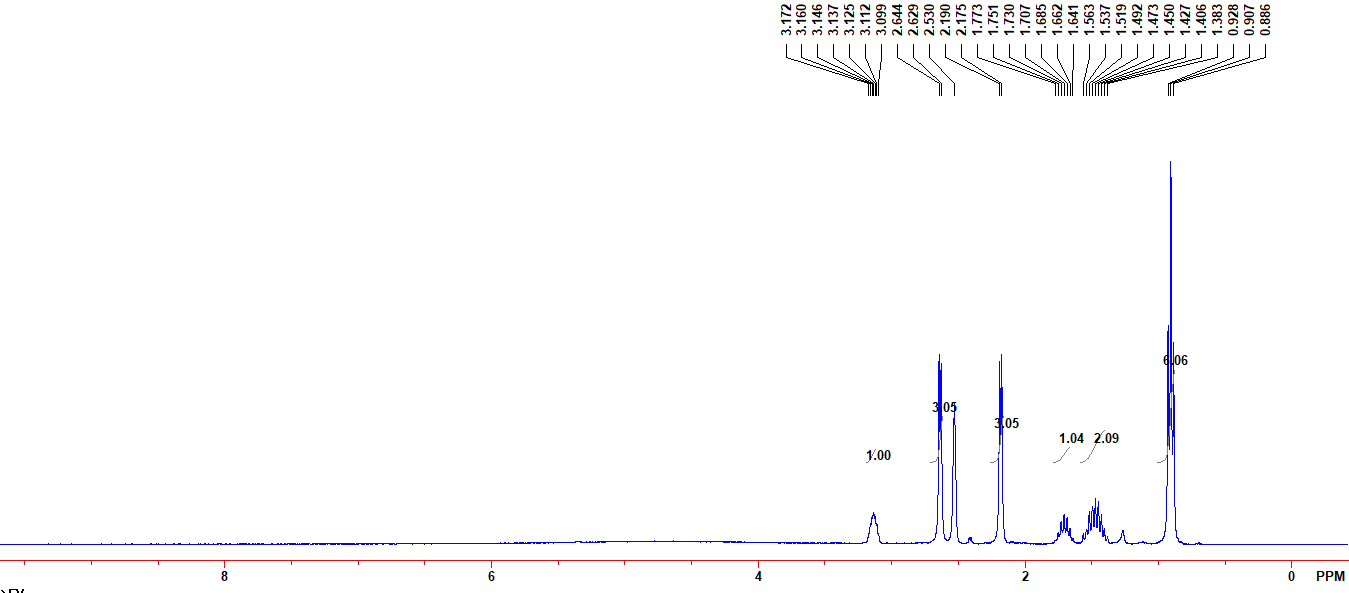
E**

**F**


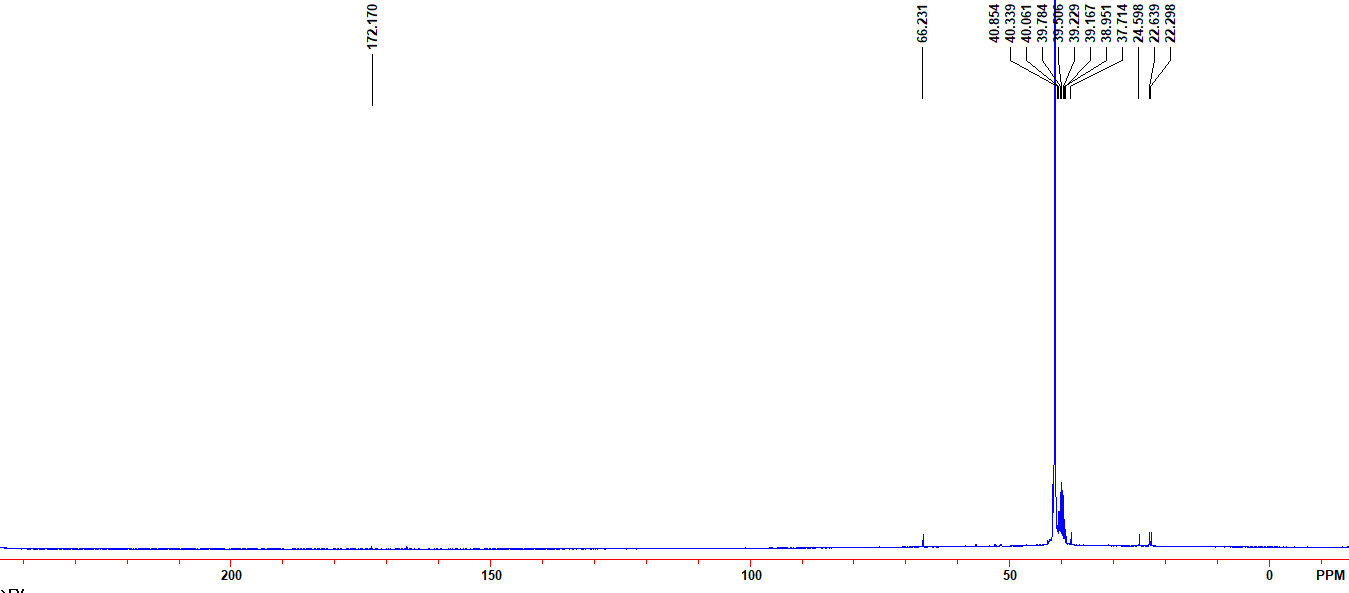

**Supplementary Figure 4.**


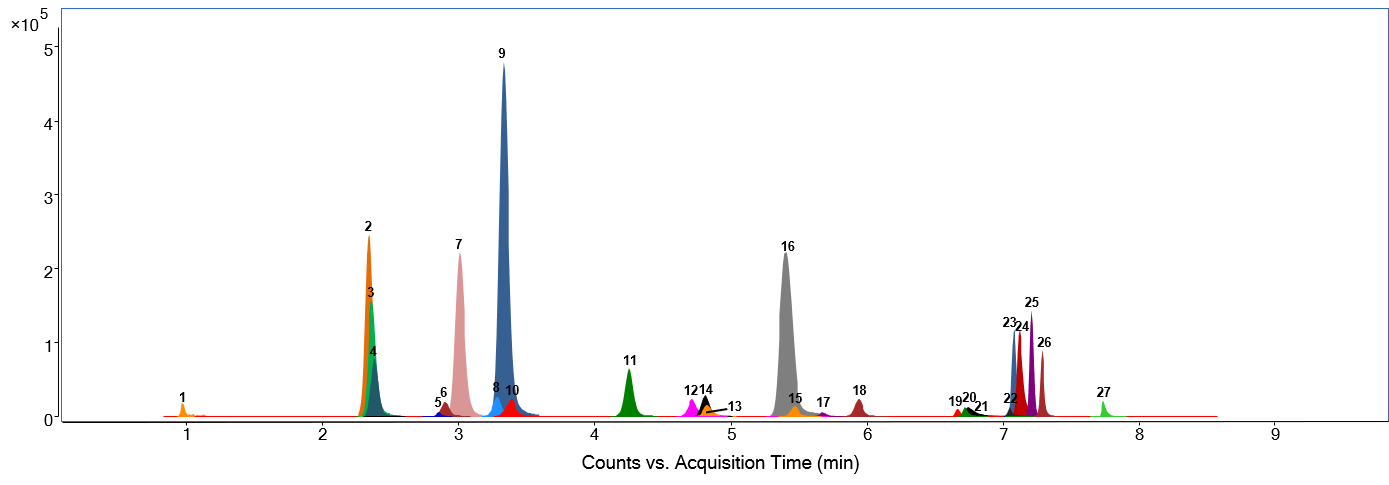


Supplementary Figure 5.


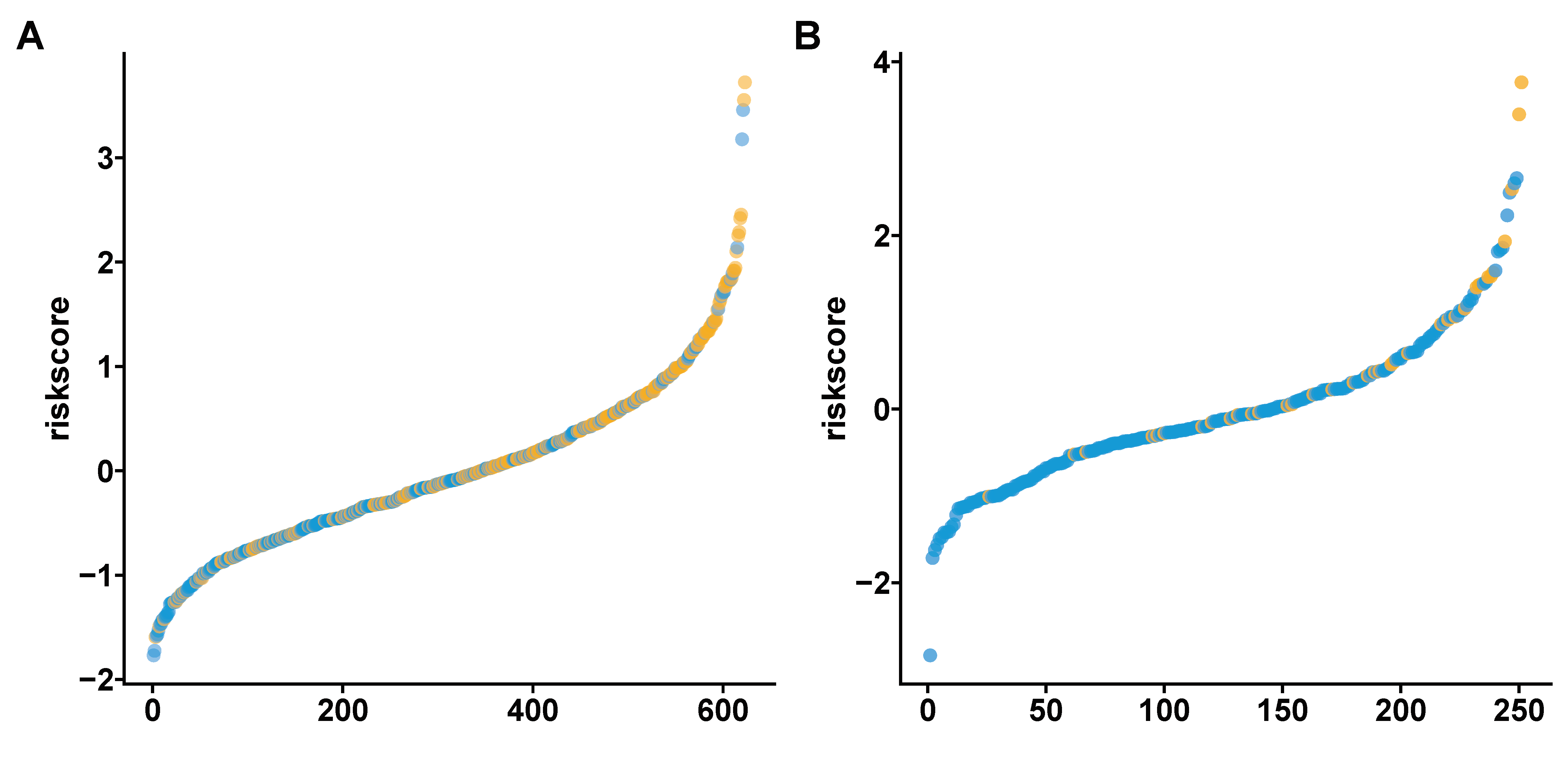


### Supplementary Figure 6.


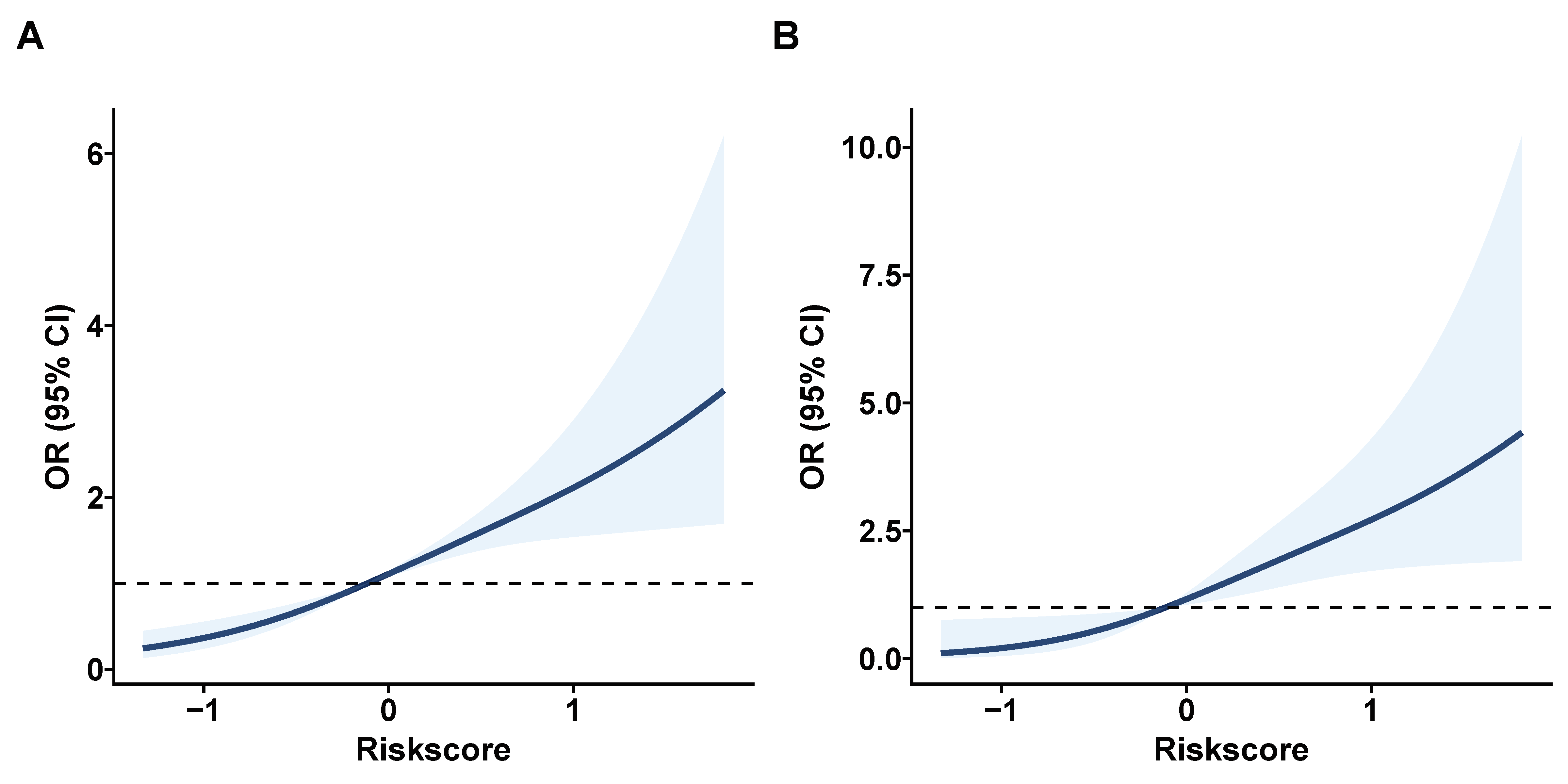


| **Amino Acids** | **Precursor Ion (*m/z*)** | **Product Ion (*m/z*)** | **Fragmentor (V)** | **Collision Energy (eV)** |
| --- | --- | --- | --- | --- |
| N,N-dimethylleucine | 160 | 114 | 100 | 10 |
| ^13^C-N,N-dimethylleucine | 162 | 116 | 100 | 10 |
| Leucine + Isoleucine | 132 | 86 | 90 | 6 |
| ^13^C_6_-L-leucine | 138 | 91 | 90 | 10 |
| Phenylalanine | 166 | 120 | 90 | 16 |
| Tryptophan | 205 | 188 | 90 | 7 |
| N-methylproline | 130 | 84 | 80 | 17 |
| ^13^C-N-methylproline | 131 | 85 | 100 | 20 |
| Methionine | 150 | 56 | 90 | 18 |
| Valine | 118 | 72 | 85 | 16 |
| N,N-dimethylglycine | 104 | 58 | 80 | 16 |
| Proline | 116 | 70 | 90 | 16 |
| Tyrosine | 182 | 91 | 90 | 32 |
| ^13^C-N,N-dimethylserine | 136 | 90 | 90 | 19 |
| Alanine | 90 | 44 | 80 | 8 |
| Threonine | 120 | 74 | 80 | 6 |
| Glycine | 76 | 30 | 80 | 16 |
| Glutamic acid | 148 | 84 | 80 | 16 |
| Glutamate | 147 | 84 | 90 | 16 |
| Serine | 106 | 60 | 90 | 12 |
| Asparaginate | 133 | 87 | 125 | 14 |
| Citrulline | 176 | 70 | 80 | 26 |
| N6-dimethyllysine | 175 | 84 | 110 | 22 |
| N6-trimethyllysine | 189 | 84 | 100 | 25 |
| ^13^C-N_5_,N_5_-dimethylornithine | 163 | 70 | 80 | 26 |
| 1-methylhistidine | 170 | 124 | 90 | 15 |
| 3-methylhistidine | 170 | 96 | 110 | 22 |
| Arginine | 175 | 70 | 110 | 26 |
| Lysine | 147 | 84 | 90 | 16 |
| Histidine | 156 | 110 | 80 | 8 |
| Ornithine | 133 | 70 | 80 | 16 |
| Cystine | 241 | 74 | 100 | 26 |

Table S1. The MS parameters of the 27 amino acids and 5 ^13^C-labeled internal standards.

Table S2. The linearity, lower limit of quantitation (LLOQ) and accuracy of the LC-MS/MS method.

| **Amino Acids** | **Internal Standards** | **Regression**  **Equation** | **Regression Coefficient** | **Range (μg/mL)** | **LLOQ (μg/mL)** |  | **Accuracy (%)** | | |
| --- | --- | --- | --- | --- | --- | --- | --- | --- | --- |
|  |  | **(1/*x*)** | **(*r*^2^)** |  |  |  | **Low** | **Middle** | **High** |
| N,N-dimethylleucine | ^13^C-N, N-dimethylleucine | y=219.67x+0.043 | 0.996 | 0.00015-0.0096 | 0.00015 |  | 105.7 | 112.0 | 92.0 |
| Leucine + Isoleucine | ^13^C_6_-L-leucine | y=0.065x+0.007 | 0.998 | 2.4-60 | 2.4 |  | 98.0 | 107.9 | 102.6 |
| Phenylalanine | ^13^C_6_-L-leucine | y=0.044x+0.001 | 0.993 | 1.2-60 | 1.2 |  | 94.2 | 93.5 | 104.7 |
| Tryptophan | ^13^C_6_-L-leucine | y=7.105x-0.791 | 0.996 | 0.6-60 | 0.6 |  | 106.8 | 97.1 | 95.0 |
| N-methylproline | ^13^C-N-methylproline | y=20.825x-0.008 | 0.992 | 0.000375-0.6 | 0.000375 |  | 111.6 | 105.6 | 85.5 |
| Valine | ^13^C_6_-L-leucine | y=0.060x+0.061 | 0.998 | 9.6-240 | 9.6 |  | 98.3 | 100.1 | 102.0 |
| Methionine | ^13^C_6_-L-leucine | y=0.473x+0.026 | 0.994 | 0.3-30 | 0.3 |  | 95.5 | 102.7 | 104.4 |
| N,N-dimethylglycine | ^13^C-N, N-dimethylleucine | y=0.098x+0.007 | 0.994 | 0.03-6 | 0.03 |  | 100.5 | 105.8 | 104.3 |
| Proline | ^13^C_6_-L-leucine | y=0.033x-0.003 | 0.995 | 1.8-90 | 1.8 |  | 102.1 | 107.1 | 106.6 |
| Tyrosine | ^13^C_6_-L-leucine | y=0.050x-0.004 | 0.993 | 3-75 | 3 |  | 97.6 | 103.0 | 106.0 |
| Alanine | ^13^C_6_-L-leucine | y=0.013x+0.003 | 0.995 | 1.2-120 | 1.2 |  | 89.5 | 99.7 | 100.4 |
| Threonine | ^13^C-N,N-dimethylserine | y=0.172x-0.141 | 0.992 | 0.75-75 | 0.75 |  | 108.2 | 92.0 | 101.0 |
| Glycine | ^13^C_6_-L-leucine | y=0.004x+0.0003 | 0.998 | 1.8-180 | 1.8 |  | 104.6 | 96.2 | 101.0 |
| Glutamic acid | ^13^C_6_-L-leucine | y=0.032x-0.009 | 0.997 | 0.3-30 | 0.3 |  | 109.0 | 91.0 | 103.0 |
| Glutamine | ^13^C_6_-L-leucine | y=0.004x+0.019 | 0.998 | 18-900 | 18 |  | 88.0 | 102.8 | 101.8 |
| Serine | ^13^C_6_-L-leucine | y=0.004x+0.0004 | 0.997 | 1.5-37.5 | 1.5 |  | 95.2 | 103.3 | 99.3 |
| Asparaginate | ^13^C_6_-L-leucine | y=0.0004x-0.0001 | 0.996 | 0.3-30 | 0.3 |  | 100.0 | 94.1 | 97.8 |
| Citrulline | ^13^C-N_5_,N_5_-dimethylornithine | y=0.021x+0.002 | 0.995 | 0.3-30 | 0.3 |  | 90.5 | 103.5 | 99.8 |
| N6-dimethyllysine | ^13^C-N_5_,N_5_-dimethylornithine | y=5.279x-0.08 | 0.996 | 0.015-1.5 | 0.015 |  | 88.7 | 102.8 | 96.2 |
| N6-trimethyllysine | ^13^C-N_5_,N_5_-dimethylornithine | y=0.398x-0.0004 | 0.992 | 0.006-0.6 | 0.006 |  | 100.8 | 92.0 | 95.6 |
| 1-methylhistidine | ^13^C-N_5_,N_5_-dimethylornithine | y=0.159x-0.003 | 0.999 | 0.015-12 | 0.06 |  | 111.2 | 99.1 | 96.8 |
| 3-methylhistidine | ^13^C-N_5_,N_5_-dimethylornithine | y=0.044x+0.001 | 0.996 | 0.00375-6 | 0.00375 |  | 105.6 | 102.9 | 111.7 |
| Arginine | ^13^C-N_5_,N_5_-dimethylornithine | y=0.03x-0.012 | 0.996 | 0.75-75 | 0.75 |  | 102.9 | 98.3 | 97.7 |
| Histidine | ^13^C-N_5_,N_5_-dimethylornithine | y=0.017x-0.002 | 0.997 | 1.2-120 | 1.2 |  | 87.5 | 106.4 | 101.5 |
| Lysine | ^13^C_6_-L-leucine | y=0.035x+0.010 | 0.999 | 0.9-90 | 0.9 |  | 120.4 | 96.7 | 101.0 |
| Ornithine | ^13^C_6_-L-leucine | y=0.095x+0.004 | 0.992 | 0.6-60 | 0.6 |  | 105.6 | 100.4 | 108.4 |
| Cystine | ^13^C_6_-L-leucine | y=0.039x-0.073 | 0.993 | 3.6-90 | 3.6 |  | 99.2 | 108.2 | 100.3 |

Table S3. Inter- and intra- day precision of the analytical method.

| **Amino Acids** | **Inter (RSD %)** | | |  | **Intra (RSD %)** | | |
| --- | --- | --- | --- | --- | --- | --- | --- |
|  | **Low** | **Middle** | **High** |  | **Low** | **Middle** | **High** |
| N,N-dimethylleucine | 2.84 | 1.64 | 1.55 |  | 1.45 | 1.18 | 1.09 |
| Leucine + Isoleucine | 2.15 | 1.53 | 2.61 |  | 1.94 | 1.27 | 0.78 |
| Phenylalanine | 3.06 | 3.93 | 1.72 |  | 3.38 | 2.48 | 0.26 |
| Tryptophan | 2.38 | 1.69 | 2.70 |  | 2.58 | 1.29 | 0.31 |
| N-methylproline | 1.39 | 2.29 | 1.54 |  | 1.00 | 1.10 | 0.53 |
| Methionine | 2.43 | 1.14 | 2.37 |  | 1.16 | 1.14 | 0.35 |
| Valine | 4.30 | 3.90 | 3.74 |  | 3.47 | 1.89 | 2.28 |
| N,N-dimethylglycine | 3.22 | 1.80 | 2.39 |  | 2.68 | 2.38 | 0.47 |
| Proline | 1.29 | 0.83 | 2.09 |  | 0.69 | 0.75 | 0.59 |
| Tyrosine | 2.34 | 1.18 | 2.24 |  | 1.99 | 0.87 | 2.58 |
| Alanine | 1.81 | 0.73 | 2.12 |  | 0.91 | 0.63 | 0.46 |
| Threonine | 2.34 | 5.24 | 3.71 |  | 2.77 | 0.78 | 0.67 |
| Glycine | 1.93 | 0.65 | 2.34 |  | 1.89 | 0.80 | 1.23 |
| Glutamic acid | 3.30 | 5.30 | 1.97 |  | 1.03 | 1.89 | 0.97 |
| Glutamine | 6.81 | 3.58 | 4.27 |  | 0.02 | 0.01 | 0.01 |
| Serine | 4.69 | 2.86 | 3.81 |  | 1.37 | 1.46 | 0.71 |
| Asparaginate | 2.65 | 2.89 | 1.58 |  | 2.65 | 2.89 | 1.58 |
| Citrulline | 4.85 | 1.22 | 4.55 |  | 1.47 | 1.41 | 1.67 |
| N6-dimethyllysine | 5.71 | 1.38 | 1.43 |  | 1.84 | 1.05 | 1.28 |
| N6-trimethyllysine | 4.96 | 1.59 | 3.07 |  | 2.67 | 1.98 | 0.58 |
| 1-methylhistidine | 6.64 | 4.41 | 5.30 |  | 3.24 | 4.93 | 7.12 |
| 3-methylhistidine | 5.65 | 5.15 | 3.16 |  | 3.59 | 7.40 | 4.45 |
| Arginine | 4.52 | 2.05 | 2.58 |  | 4.27 | 2.63 | 1.61 |
| Lysine | 0.96 | 1.43 | 1.81 |  | 2.00 | 1.20 | 0.54 |
| Histidine | 2.87 | 3.15 | 1.08 |  | 4.12 | 3.83 | 0.70 |
| Ornithine | 1.79 | 2.45 | 2.38 |  | 1.51 | 3.20 | 0.66 |
| Cystine | 5.99 | 4.97 | 5.35 |  | 2.74 | 5.22 | 2.76 |

Table S4. The C-indices of base model and amino acid-assisted model.

| **Centers** | **Base Model**  **(C-Indices)** | **Amino Acid-Assisted Model**  **(C-Indices)** |
| --- | --- | --- |
| Center 1 | 73.87% (95% CI: 69.71%-78.04%) | 76.51% (95% CI: 72.56%-80.47%) |
| Center 2 | 82.05% (95% CI: 72.14%-91.97%) | 89.58% (95% CI: 82.76%-96.40%) |
